# Extreme lateral interbody fusion (XLIF) approach for L5-S1: Preliminary experience

**DOI:** 10.3389/fsurg.2022.995662

**Published:** 2022-09-27

**Authors:** Junjie Xu, Enliang Chen, Le Wang, Xiaobao Zou, Chenfu Deng, Junlin Chen, Rencai Ma, Xiangyang Ma, Zenghui Wu

**Affiliations:** ^1^ The First School of Clinical Medicine, Southern Medical University, Guangzhou, China; ^2^ Department of Orthopedics, General Hospital of Southern Theatre Command of Chinese PLA, Guangzhou, China; ^3^ Department of Orthopedics, Foshan Hospital of Traditional Chinese Medicine, Foshan, China; ^4^ Department of Orthopedics, Third Affiliated Hospital of Guangzhou Medical University, Guangzhou, China

**Keywords:** XLIF, L5-S1, iliac crest, iliac vessels, lumbar plexus, surgical technique

## Abstract

**Study Design:**

Technical report.

**Objective:**

Evaluate technical feasibility of extreme lateral interbody fusion (XLIF) at the L5-S1 level and provide an elaborate description of the surgical technique.

**Summary of Background Data:**

With the development of surgical techniques, the indications for oblique lumbar interbody fusion (OLIF) surgery have been broadened to the L5/S1 segment. However, this technique also has limitations. Different from OLIF, the L5/S1 segment used to be considered the main contraindication for XLIF. To date, no authors have reported the application of XLIF at the L5/S1 level.

**Methods:**

Only patients whose preoperative lumbar MRI showed the position of the psoas major muscles and blood vessels at the L5/S1 level were similar to those seen at supra-L5 levels were seleted. By folding the operating table, the iliac crest was moved downward to expose the L5/S1 intervertebral space during the operation. The remaining surgical procedures were consistent with routine XLIF surgery.

**Results:**

8 patients successfully underwent XLIF at the L5/S1 level. The L5/S1 disk spaces were always exposed sufficiently for disk preparation and cage insertion. The post operative radiographs showed a satisfactory L5/S1 reconstruction with good cage position. Only 1 patient (12.5%) felt thigh numbness, and the symptoms gradually resolved after surgery and were no longer present in a month. There were no cases of psoas hematoma, retrograde ejaculation or vascular injury. The postoperative VAS score showed that all the patients achieved satisfactory results.

**Conclusions:**

XLIF at L5-S1 is feasible in strictly selected cases after thorough preoperative preparation and careful intraoperative procedures. However, we did not recommend XLIF as a routine surgical option at the L5/S1 level.

## Introduction

Over the past decades, improvements in surgical techniques and spinal instrumentation have allowed surgeons to develop safe and solid constructs for various degenerative lumbar diseases, such as degenerative disc disease, spondylolisthesis and deformities (1). To avoid vascular and visceral risks associated with anterior approaches (2, 3) and neural complications and bony resection common to posterior approaches (4, 5), extreme lateral interbody fusion (XLIF) and oblique lumbar interbody fusion (OLIF) were developed as less-invasive alternatives, with the advantages of less blood loss and operative time, higher fusion rates and more satisfactory clinical results.

With the further development of surgical techniques, the indications for OLIF surgery have been broadened to the L5/S1 segment. Some surgeons perform OLIF between the bifurcations beneath the iliac vessels, which is actually a lateral decubitus ALIF (1). Similar to the traditional OLIF procedure for L2-L5, another surgeon reached the intervertebral spaces of L5/S1 between the iliac vessels and the psoas, which also achieved satisfactory clinical results (6, 7). However, this technique also has limitations.

In a meta-analysis of 1874 oblique lumbar interbody fusion patients, Walker et al. (8) found that the main complications were sympathetic plexus injury and major vascular injury, with risk rates of 5.4% and 1.8% respectively. Other studies also suggest a higher risk of vascular injury in patients who undergo OLIF at L5-S1 compared to those who undergo ALIF at L5-S1 (6), or OLIF at supra-L5 levels (9).

Different from OLIF, the L5/S1 segment used to be considered the main contraindication for XLIF. It is generally believed that the extremely lateral approach to L5-S1 is extremely difficult to create because of the presence of the iliac crest, the iliac vessels and the location of the psoas muscle. To date, no authors have reported the application of XLIF at the L5/S1 level.

To further expand the application of XLIF at the L5/S1 level and avoid the complications associated with vessel separation related to OLIF, we attempted to perform discectomy and fusion at the L5-S1 level, including other levels, using XLIF techniques. This study confirmed that XLIF could be applied in strictly selected patients through a small sample research.

## Materials and methods

### Inclusion and exclusion criteria

This was a retrospective study. Inclusion criteria were L5 degenerative spondylolisthesis (Grade I or II), degenerative lumbar scoliosis and degenerative lumbar kyphosis confirmed by imaging, with varying degrees of low back or lower extremity pain, with neurological dysfunction such as weakness in 1 or both the lower extremities. Exclusion criteria were L5 spondylolisthesis (degree III and above), history of anterior or posterior lumbar surgery, lumbar trauma, infection, tumor, severe osteoporosis, and history of abdominal surgery within the previous year.

### Surgical techniques

Thorough preoperative preparation was crucial. First, confirming the position of the iliac crest in the preoperative standing lateral radiograph. If the iliac crest was below the 1/2 line of the L5 vertebra, then the L5/S1 intervertebral space could be exposed when the iliac crest was moved downward by folding the operating table. Second, make sure the iliac vessels were located in the first quarter or more ahead of the intervertebral space, and at least 2/3 of the psoas major muscles are located on the lateral side of the vertebral body. Last, in order to protect the neurovascular structures in the cleft which was identified between the psoas major and the ipsilateral L5/S1 intervertebral space, make sure the distance from the iliac vessels to the neural structures in the cleft was beyond a quarter of the ipsilateral L5/S1 intervertebral space in the preoperative MR picture.

Careful intraoperative procedures also played an important role. All procedures were performed by one spine surgeon with XLIF experience. Under general anesthesia, patients were placed in a true lateral position with the top hip and knee flexed. After folding the operative table, fluoroscopy confirmed that the L5/S1 intervertebral space was exposed without obstruction of the iliac crest. Then, an incision was cut in the center of the surface projection of the L5/S1 intervertebral disc. Once the incision was made, blunt dissection of the external and internal oblique muscle, transverse abdominal muscle, and transverse fascia was performed. Then, the surgeon could reach the retroperitoneal space with a finger. By pushing the peritoneal tissue forward, we can reveal the shape of the psoas major. Then the psoas major muscle was bluntly split layer by layer in the first third part. After confirming that there were no blood vessels or nerve structures in the lateral side of the intervertebral space, the expansion channel could be placed. Then, discectomy was performed. Importantly, the contralateral annulus was not excised to prevent the cage from injuring the contralateral psoas, vessels and nerves. Then, a polyetheretherketone (PEEK) cage filled with allograft bone and autologous bone marrow, which were extracted from the vertebral body, was implanted. If the BMD T score of a patient was equal to or greater than −2.5, then the surgeon would select unilateral pedicle screw for fixation through the Wiltse approach in the same position. If the BMD T score of a patient was less than −2.5, then the surgeon would prefer to use bilateral pedicle screws for fixation through the Wiltse approach by changing positions during surgery.

## Results

After our detailed evaluation and planning, 8 patients successfully underwent XLIF at the L5-S1 level. Among them, 4 patients were diagnosed with degenerative lumbar scoliosis, 3 with lumbar spondylolisthesis, and 1 with degenerative lumbar kyphosis (Table 1). Due to the different surgical levels, the operative time and blood loss of each patient varied greatly, but they were basically discharged from the hospital approximately one week after surgery.

**Table 1:** Patient demographics of the surgery.

Case	Sex	Age (y)	Diagnosis	XLIF Level	Cage Height (mm)	OR time	EBL	LOS
1	F	60	Degenerative lumbar scoliosis	L2/3-L5/S1	10	357	200	7
2	F	47	L5 degenerative spondylolisthesis (Grade II)	L5/S1	12	120	100	6
3	F	66	Degenerative lumbar scoliosis	L2/3-L5/S1	7	407	450	8
4	M	58	L5 degenerative spondylolisthesis (Grade I)	L5/S1	9	180	200	8
5	F	56	L5 degenerative spondylolisthesis (Grade I)	L5/S1	10	200	100	5
6	F	70	Degenerative lumbar scoliosis	L2/3-L5/S1	8	427	220	9
7	F	59	Degenerative lumbar scoliosis	L3/4-L5/S1	12	350	300	8
8	F	67	Degenerative lumbar kyphosis	L1/2-L5/S1	10	450	350	9

F, female; M, male; OR time, operative time(min); EBL, estimated blood loss (ml); LOS, length of stay (d).

Segmental lordosis and disk height significantly increased after the operation (Table 2). Only 1 patient (12.5%) felt thigh numbness, and the symptoms gradually resolved after surgery and were no longer present in a month. There were no cases of psoas hematoma, retrograde ejaculation or vascular injury. The postoperative VAS score showed that all the patients achieved satisfactory results.

**Table 2:** Radiographic results and complications of the surgery.

Case	Pre- DH	Post- DH	Pre- SL	Post-SL	Pre- VAS	Post- VAS	Complication
1	5.00	9.75	7.64	16.92	7	3	/
2	4.70	11.94	18.29	18.73	8	2	/
3	3.92	6.91	4.62	14.54	7	4	/
4	7.61	8.98	16.47	21.09	6	2	/
5	7.24	9.50	6.96	7.97	6	2	/
6	4.76	7.74	12.56	13.86	8	3	Thigh numbness
7	5.60	11.78	14.69	18.98	6	1	/
8	6.20	9.31	16.88	19.53	7	2	/

Pre- DH, preoperative disc height(mm); Post- DH, postoperative disc height(mm); Pre- SL, preoperative segmental lordosis (°); Post-SL, postoperative segmental lordosis (°); Pre- VAS, peroperative visual analogue scale; Post-VAS, postoperative visual analogue scale.

### Case presentation 1

**Figure 1:**
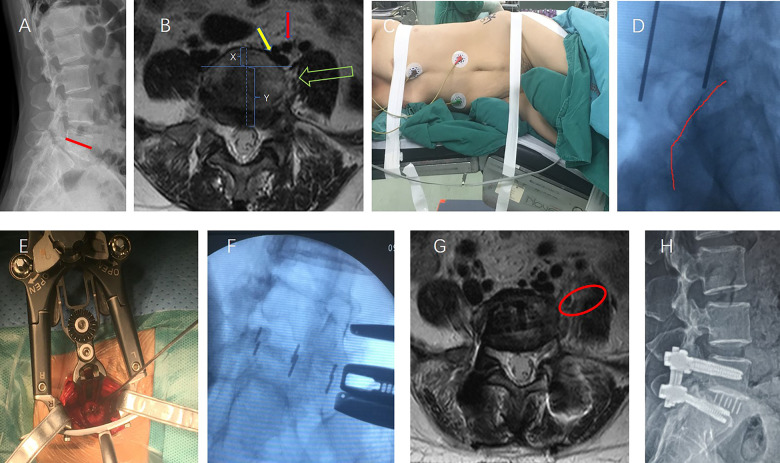
A 47-year-old woman underwent XLIF at the L5/S1 level. (**A**) Preoperative radiograph showed L5 degenerative spondylolytic spondylolisthesis, and the iliac crest was below the 1/2 line of the L5 vertebra (the red line). (**B**) The picture shows that the XLIF approach at L5-S1 in this case (green arrow) was feasible without obstruction of the left common iliac vein (yellow arrow) and the left iliac artery (red arrow). The iliac vessels were located in the first quarter of the intervertebral space (X:Y < 1:3). (**C**) Surgical position. (**D**) The iliac crest (red line) is lowered below the L5/S1 intervertebral space. (**E**) Intraoperative photograph showing the L5–S1 disk was exposed. (**F**) Intraoperative fluoroscopy after the implantation of the cage. (**G**) The red circle shows the working channel through the left psoas at L5/S1. (**H**) Radiographs taken 3 months postoperatively showed slip reduction with good cage positioning.

A 47-year-old woman presented with a 20-year history of low back pain, and a 5-year history of radiating pain in the left lower extremity. Her symptoms worsened in the past 2 years and she was able to walk for only 20 meters. There was no apparent motor weakness or sensory disturbance in the lower extremities. Her standing lateral radiograph showed L5 spondylolytic spondylolisthesis, and the iliac crest was below the 1/2 line of the L5 vertebra (Figure 1A). In addition, the locations of the psoas major muscle and blood vessels in the preoperative MR images picture were consistent with those seen in routine XLIF surgery. The iliac vessels were located in the first quarter of the intervertebral space, and the psoas major muscle was close to the ipsilateral L5/S1 intervertebral space and did not move forward.

Thus, we decided to perform XLIF (NuVasive,Inc.) at the L5/S1 level. The total surgical time was 120 min, and the blood loss volume was 100 ml. The patient was symptom-free and able to walk just 1 day after the surgery. The patient was discharged from the hospital on the sixth day. There were no perioperative complications during the surgical access and reconstruction procedures.

### Case presentation 2

A 56-year-old woman presented with an 8-month history of radiating pain and numbness in the left aspect of her buttock, back of the thigh and back of the calf. Her symptoms worsened in the past 1 month, and she was unable to walk. There was no apparent motor weakness in the lower extremities. The sensory examination confirmed hypoalgesia in the left aspect of her posterior shank, pedis and pelma corresponding to the L5 and S1 dermatomes. The preoperative lumbar x-ray examination showed L5 degenerative spondylolisthesis.

**Figure 2:**
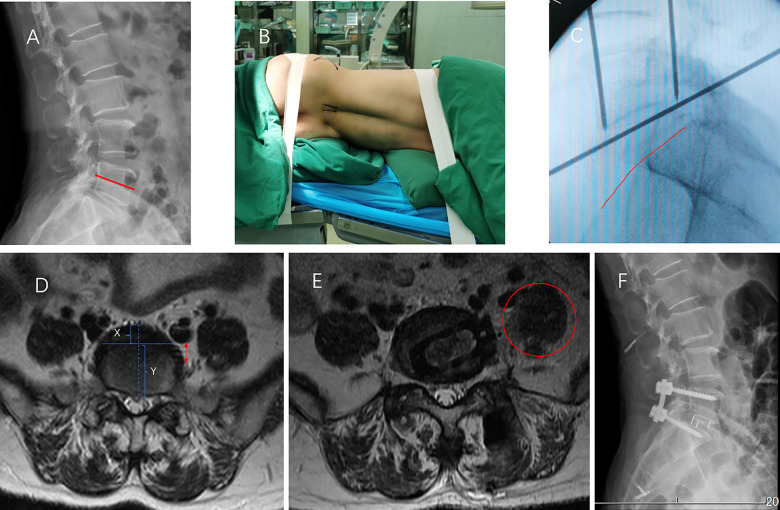
A 56-year-old woman underwent XLIF at the L5/S1 level. (**A**) Preoperative radiograph showed L5 degenerative spondylolytic spondylolisthesis, and the iliac crest was below the 1/2 line of the L5 vertebra (the red line). (**B,C**) The iliac crest (**C** red line) is lowered below the L5/S1 intervertebral space after folding the operating table (**B**). (**D**) The iliac vessels were located in the first quarter of the intervertebral space(X:Y < 1:3). There is no obvious nerve or vascular tissue between the psoas and the lateral side of the intervertebral space. (**E**) The red circle shows the working channel through the left psoas at L5/S1. (**F**) Radiographs taken at 3 days postoperatively showed a good cage position.

The iliac crest of this case was also below the 1/2 line of the L5 vertebra (Figure 2A). In this CASE, although the locations of the psoas major muscle and blood vessels in the preoperative MR picture were similar to those seen in Case 1, a cleft containing loose connective tissue and neurovascular structures was identified between the psoas major and the ipsilateral L5/S1 intervertebral space. These special structures may increase the risk of XLIF surgery. However, the distance from the left iliac vessels to the neural structures in the cleft (Figure 2D. Red double-headed arrow) was beyond a quarter of the ipsilateral L5/S1 intervertebral space in the preoperative MR picture. Based on the above anatomical basis, we believe that this distance may meet the safe space required for the XLIF operation.

After detailed planning, we successfully performed XLIF (DePuy Synthes) at L5-S1 (using a PEEK cage, DePuy Synthes, Raynham, MA, USA) followed by posterior L5 to S1 pedicle screw placement unilaterally with the Wiltse approach in the same position (Figure 2). The total surgical time was 100 min, and the blood loss volume was 100 ml. The patient was symptom-free and able to walk just 1 day after the surgery. The patient was able to be discharged from the hospital on the fifth day. There were no perioperative complications during the surgical access and reconstruction procedures.

## Discussion

Since Ozgur et al. (10) first described extreme lateral interbody fusion (XLIF) in 2006, this less invasive alternative to conventional anterior and posterior approaches for interbody fusion has been used. Similar to the anterior approach for lumbar interbody fusion, the lateral approach allows a wide-footprint intervertebral cage with wide apertures to be placed to provide superior anterior column realignment as well as a healthy fusion environment without the need for anterior and posterior longitudinal ligament resection (11–13).In our prior study, we performed XLIF in 107 patients at 126 levels to examine the subsidence and fusion rate after 2 years. The results were satisfactory, and moreover, the rate of injury to the neural structures of the lumbar plexus was relatively low (14). However, these results did not include those at the L5-S1 level.

With improvements in surgical techniques and spinal instrumentation, various minimally invasive surgical methods, such as anterior lumbar interbody fusion(ALIF), oblique lumbar interbody fusion (OLIF) and anterior to psoas (ATP) approaches, have been performed by surgeons to treat lumbar diseases at the L5-S1 level (15, 16). The anterior retroperitoneal approach in ALIF facilitates adequate access to the entire ventral surface of the exposed disc, allowing comprehensive discectomy and direct implant insertion as well as sparing of posterior spinal muscles and anterolateral psoas muscles, which may reduce postoperative pain and disability (17). However, ALIF involves complications such as abdominal visceral injury, anterior lumbar vascular injury, retrograde ejaculation, intestinal adhesion and abdominal hernia (18, 19). A recent study reported that retrograde ejaculation occurred in 7.4% of cases and vascular injury occurred in 6.1% of cases (8, 20).

In a study of 179 oblique lumbar interbody fusion (OLIF) patients, Silvestre (21) suggested that another approach might be preferred at L5-S1 due to the risks associated with mobilization of the iliac vessels and the presence of the iliac wing. Different from OLIF, the ATP approach reaches the surgical field between the iliac vessels and the psoas, requiring a dedicated surgical retractor and an advanced operating technique not only for the L2–L5 segments but also for the L5/S1 segment. This approach avoids injury to the lumbar plexus and has a greater ability to correct lordosis and improve alignment (22). On the other hand, Wei He et al. (23) recommend that the most difficult aspect of the ATP approach is securing with vascular structures around the L5/S1 disc, and surgeons should identify all relevant vessels and either secure or ligate them during surgery. We believe that this manipulation of the blood vessels may cause unnecessary damage and sometimes disastrous consequences.

Because of the obstruction of the iliac crest, the L5/S1 segment used to be considered the main contraindication for XLIF. In the selected cases in which we performed XLIF at the L5/S1 level, the iliac crest was relatively low and we could easily reach the intervertebral space from the lateral side after folding the operative table. In addition, we suggested choosing the appropriate cases in which the psoas was close to the lateral side of the L5/S1 intervertebral space and the vascular structures around this level were relatively forward to perform XLIF to reduce vascular-related complications through the operative channel established by blunt dissection of the psoas. The most common complication of XLIF was injury to the lumbar plexus. We need to have a thorough understanding of the nerve tissues around L5-S1 to avoid related complications. *The femoral nerve, the obturator nerve and the genitofemoral nerve* are the three branches of the lumbar plexus that were carefully avoided during the surgery. According to Uribe et al. (24, 25), *the femoral nerve*, formed from branches of the L2, L3, and L4 roots, was found deep in the psoas muscle, descending in a gradual posterior-to-anterior trajectory at the L4–5 disc space and continuing downward between the psoas and the iliacus muscle. In a study by Jianfei Ji et al. (26), *the femoral nerve* of all 6 specimens was located at the posterior middle quarter and the posterior quarter part at the L5-S1 level. *The genitofemoral nerve* travels obliquely in the psoas muscle from its origin, crosses the L2–3 disc space, and emerges from its medial border superficial and anterior at the L3–4 level, then lies on the anterior surface of the psoas at the L4–5 level, and finally descends along the surface of the psoas major (24). *The obturator nerve,* as one branch of the lumbar plexus, passes obliquely through the cleft of the psoas major, from the posterior border of the L4 vertebrae to the anterior border of the L5/S1 disc (26). Therefore, the probability of injury to *the genitofemoral nerve* and *the obturator nerve,* which are often located at the anterior quarter part of the psoas at the L5/S1 level, as well as the *femoral nerve,* which is located at the posterior half part of the psoas at the L5/S1 level*,* would be reduced when placing an operating corridor through the middle anterior quarter of the psoas and retracting the corridor under direct vision.

Based on our thorough preoperative preparation and careful intraoperative procedures, the operative channels through the psoas at the L5/S1 level were successfully established in a total of 8 patients, although we had prepared OLIF tools ready to switch to OLIF if the XLIF channel was difficult to create. Therefore, we concluded that XLIF could be performed at the L5/S1 level when the following five requirements are met: (1) the highest point of the iliac crest is located below the 1/2 line of the L5 vertebral body, (2) the iliac vessels are located in the first quarter or more ahead of the intervertebral space, (3) at least 2/3 of the psoas major muscles are located on the lateral side of the vertebral body, (4) there is no obvious nerve and vascular tissue between the psoas and the lateral side of the intervertebral space, and (5) the distance from the iliac vessels to the neural structures in the cleft was beyond a quarter of the ipsilateral L5/S1 intervertebral space in the preoperative MR picture. However, because of the tiny sample of the study, we suggested not to perform XLIF at L5/S1 segment unless all five indicators were met.

Only 1 patient felt thigh numbness and this symptom gradually resolved in a month. Although the probability and degree of lumbar plexus injury were mild in this study, we still considered that care should always be taken to avoid injury to the lumbar plexus. Moreover, there were no cases of psoas hematoma, retrograde ejaculation or vascular injury.

Conventional PLIF and TLIF surgery require extensive stripping of the paraspinal muscles and lead to an extensive exposure range, intense trauma, substantial bleeding and severe damage to the lumbar biomechanical structure and function, resulting in complications such as slow postoperative recovery, back stiffness, and chronic back pain. Using a transpsoas approach, XLIF of L5/S1 segment could restore the disk height and sagittal alignment can be restored by implanting a large cage without disrupting the back muscles, anterior and posterior longitudinal ligaments, or facet joints. Therefore, XLIF of L5/S1 segment has its advantages of less tissue trauma and postoperative pain, shorter hospital stays and quicker recovery. Compared with ALIF, XLIF at L5-S1 has a lower risk of abdominal visceral injury, anterior lumbar vascular injury, retrograde ejaculation, intestinal adhesion, and ventral hernia. Compared with OLIF, XLIF has a lower risk of vascular injury. At the same time, the XLIF cage is larger than the OLIF cage, which can span the entire cortical ring, and the risk of postoperative vertebral space collapse is reduced. Compared with ATP, XLIF surgery is not associated with a higher risk of injury to the sympathetic chain. Finally, we suggest that XLIF surgery at the L5/S1 segment is feasible in certain cases, but it can not be recommended as a routine surgical option.

### Limitations

The study has certain limitations. Firstly, this was a retrospective study with a tiny sample and potential selection biases. Secondly, although we have accumulated plenty of experience in XLIF surgery, this is our first attempt to apply this technique to the L5-S1 segment. Thus, a potential technique bias may exist. Lastly, we did not have the follow-up plan because our focus was on approach-related complications that are often evident in the early postoperative period, so it is only a clinical assessment of a technique that needs to be tested.

## Conclusion

From this study, it can be concluded that XLIF at L5-S1 is feasible in strictly selected cases after thorough preoperative preparation and careful intraoperative procedures. The radiographic and clinical results were satisfactory. However, we did not recommend XLIF as a routine surgical option at the L5/S1 level. The sample size of this research was relatively small, and additional studies with longer follow-up periods are needed to demonstrate the clinical effectiveness.

## Data Availability

The raw data supporting the conclusions of this article will be made available by the authors, without undue reservation.
